# Joint Image Encryption and Screen-Cam Robust Two Watermarking Scheme

**DOI:** 10.3390/s21030701

**Published:** 2021-01-20

**Authors:** Weitong Chen, Na Ren, Changqing Zhu, Anja Keskinarkaus, Tapio Seppänen, Qifei Zhou

**Affiliations:** 1 Key Laboratory of Virtual Geographic Environment, Nanjing Normal University, Ministry of Education, Nanjing 210023, China; 171301018@njnu.edu.cn (W.C.); 09322@njnu.edu.cn (C.Z.); 181301014@njnu.edu.cn (Q.Z.); 2 State Key Laboratory Cultivation Base of Geographical Environment Evolution, Nanjing 210023, China; 3 Jiangsu Center for Collaborative Innovation in Geographical Information Resource Development and Application, Nanjing 210023, China; 4 Physiological Signal Analysis Team, Center for Machine Vision and Signal Analysis, University of Oulu, 90014 Oulu, Finland; Anja.Keskinarkaus@oulu.fi (A.K.); Tapio.Seppanen@oulu.fi (T.S.)

**Keywords:** image encryption, chaotic mapping, screen-cam process, robust watermarking, discrete fourier transform, smartphone

## Abstract

This paper proposes a joint encryption and screen-cam robust watermarking scheme. This method combines the advantages of smartphone, encryption and watermarking technologies, thereby achieving watermark extraction with a smartphone, partial decryption and tracking leakage from sneak shots. We design a dual watermarking algorithm to achieve watermark detection from both encrypted and decrypted images. First, a watermark is embedded in the discrete Fourier transform (DFT) domain to enable leakage tracking. Then, a second watermark is generated based on QR (Quick response) code encoding and inverse DFT to achieve high watermark capacity and error correction ability, where the secret key for decryption is included in the watermark message. By hiding this message carrying the watermark for the encrypted image in the changes caused by embedding the first watermark, we can improve imperceptibility and will not affect the effectiveness of the proposed scheme. Finally, to enhance the robustness of watermark after encryption, a chaotic mapping-based segment encryption algorithm is proposed. In the process of watermark detection, to cope with perspective correction, a frame locating based algorithm is employed to achieve watermark synchronization from a recaptured picture of the encrypted image. Considering the severe quality degradation, we use a noise component and local statistic feature-based method to extract the message bits. The experimental results show that the proposed scheme is secure, and highly robust, to screen-cam the process for both before and after decryption. Additionally, after decryption, the proposed scheme also has high robustness against common image processing attacks.

## 1. Introduction

With the continuous improvement of smartphone hardware and mobile applications, the functions of smartphones have become quite powerful. Nowadays, smartphones have become indispensable in our daily life. At the same time, information leakage by taking photos with a smartphone has become more common. To protect image data from being leaked, there are two typical solutions. One solution is to encrypt image data and decrypt it with a secret key when using it [1,2,3,4]. The other solution is access control technology [5,6,7,8], which prevents unauthorized access to the data. Although these methods can keep the image unreadable or inaccessible, they cannot prevent users from leaking the decrypted image displayed on the screen by taking photos with smartphones.

In order to protect image and tracking leakage, a joint encryption and watermarking scheme is an effective solution. Furthermore, smartphones are a double-edged sword in data protection. In addition to stealing data through shooting with smartphones, smartphones also have unique advantages in user identity authentication. Therefore, how to combine the advantages of smartphone, encryption and watermarking technologies for identity authentication, key management and leakage tracking is a meaningful issue.

**Figure 1:**
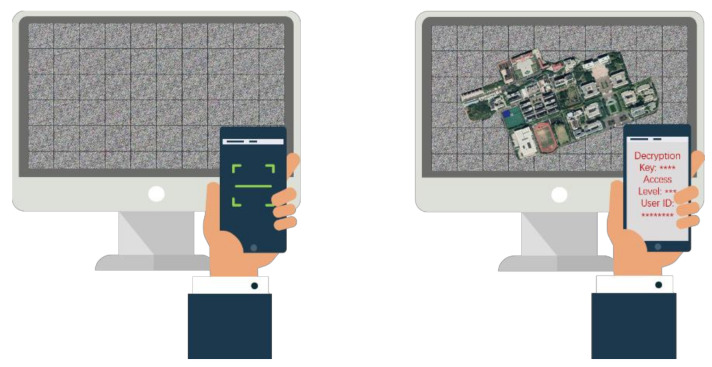
Application scenario of smartphone-based watermark reading and partial decryption.

Joint encryption and screen-cam robust watermarking has two typical application scenarios. (1) Smartphone-based message reading and partial decryption. This scenario is like reading a QR code with a mobile phone. As shown in Figure 1, we can read a secret key, access level, recipient ID and other information from the encrypted image through scanning or shooting with a smartphone and perform identity authentication based on the mobile security application. Unauthorized users will not obtain the decryption key, and authorized users will be returned with a decryption sequence that indicates the secret key and the user’s access level. Corresponding partial decryption according to the user’s access level can be performed after entering the decryption sequence in the PC software. (2) Leakage tracking. In an access control environment, it is difficult for unauthorized users to steal the data. However, authorized users can take a photo of a decrypted image to cause leakage. Once the data has been leaked through the photo, we can extract the watermark information from the photo. After that, we can locate the receiver of this data, so as to achieve accountability.

The existing researches of joint encryption and watermarking schemes mainly focus on two categories: commutative encryption and watermarking (CEW) [9,10,11,12,13,14,15] and reversible data hiding in encrypted images (RDH-EI) [16,17,18,19,20,21,22,23,24,25,26,27,28]. CEW achieves mutual independence of encryption and watermarking. RDH-EI aims to achieve lossless recovery of the original image, which is mainly designed for situations in which permanent distortion is strictly forbidden. However, due to the different purposes of the algorithm design, these schemes are designed to be robust to common image processing attacks or fragile watermarking, which means they are not applicable for the screen-cam process.

Screen-cam processing is using a camera device to regenerate the content displayed on the screen into digital signals. Hence, the screen-cam process can be considered as a cross-media signal transmission process containing digital-to-analog and analog-to-digital conversion. Similar cross-media signal transmission includes the print-scan process and print-cam process. Existing research on print-scan or print-cam robust image watermarking can be divided into three categories: watermark pattern-based methods [29,30,31,32,33,34,35], Fourier domain-based methods [36,37,38,39] and multidomain-based methods [40,41,42]. Although the ideas of these methods are valuable for studying screen-cam robust watermarking algorithms, these methods are not applicable for the screen-cam process [43]. The screen-cam process has its particularity, which causes various types of distortions [43,44,45], including linear distortion, gamma tweaking, geometric distortion, moiré noise and low-pass filter attack. To cope with these severe distortions, Fang et al. [43] proposed a feature-based watermarking scheme where the message is embedded in the discrete cosine transform (DCT) domain of local feature regions. To further improve the robustness, Fang et al. [46] proposed a deep learning-based watermarking scheme. To achieve blind detection under geometric distortion, Chen et al. [44] designed a watermark synchronization method and embedded the message in the discrete Fourier transform (DFT) domain. These methods are effective for screen-cam attack. However, to be able to detect a watermark from encrypted and watermarked images, we need to study new watermarking schemes and investigate matching encryption algorithms.

Chaos-based image encryption algorithms have been extensively researched [47,48,49,50,51,52,53] because of the advantages of chaotic system, which include high sensitivity to initial conditions and control parameters and pseudorandom behaviors [3,49]. Typical chaotic map-based encryption has two stages: permutation and diffusion [54]. Permutation operation changes the pixel positions commonly based on a generated chaotic order. Diffusion operation encrypts pixel values based on a generated chaotic sequence or matrix. For example, XOR operation is a wildly used diffusion method [55,56,57]. However, these methods are not robust to cropping attack, which means they cannot achieve partial decryption. Especially for high-resolution satellite images and secret raster maps, when facing users with different access levels, performing corresponding partial decryption is a practical and meaningful function.

As the existing joint encryption and watermarking schemes do not consider the screen-cam process, to solve this issue, a joint encryption and screen-cam robust two watermarking scheme is proposed. Furthermore, a joint encryption and watermarking scheme should not be a simple superposition of two technologies. When utilizing both technologies, they should complement each other. Similarly, how to combine the two watermarking is also important. Therefore, balancing imperceptibility and robustness while employing two watermarking, improving watermark capacity, and achieving mutual cooperation of encryption and watermarking technologies are our research objectives. The main contributions are as follows:

- We propose a dual watermarking algorithm to achieve watermark detection from both encrypted and decrypted images. Additionally, to improve imperceptibility and guarantee effectiveness of the proposed scheme, we hide the watermark for the encrypted image into the changes caused by embedding the watermark for the decrypted image.
- We design a QR (Quick Response) code encoding and inverse discrete Fourier transform (IDFT) based watermark generation method, which can improve watermark capacity and error correction ability.
- We propose a chaotic mapping-based segment encryption algorithm to cooperate with the watermarking algorithm. By applying this, the watermark can be enhanced after encryption, thereby achieving watermark extraction from the encrypted image with smartphones.

In the rest of the paper, the proposed method is introduced in Section 2. Section 3 analyzes the selection of parameters and experiment results. Section 4 gives the discussions, and Section 5 draws the conclusions.

## 2. Proposed Method

### 2.1. Embedding and Encryption Scheme

In order to achieve screen-cam robust watermarking both before and after encryption, we propose a dual watermark method and a chaos-based encryption method. With regard to watermarking algorithm, we embed watermark A and watermark B in the host images, where watermark A works in the decrypted image and watermark B works in the encrypted image. In other words, watermark A is designed for leakage tracking that can be detected from a recaptured image of a decrypted host image, and watermark B is designed for real-time information reading from a recaptured image of an encrypted host image. The key to a watermarking algorithm is to design a high capacity and error correction watermark B generation method and ensure imperceptibility by designing embedding methods. Furthermore, to achieve secure key management, the key for decryption is included in the watermark B message. Therefore, we do not need additional transmission of the secret key as separate data. With respect to the embedding region, considering the advantages of DFT domain in the screen-cam process [44], we employ DFT-based methods to embed both watermark messages in blocks repeatedly. With regard to the encryption algorithm, it is not only to achieve encryption but also to work with the watermark B. The main idea of encryption is using an odd-even segment encryption method to work with the odd-even quantization based watermarking method to achieve the purpose of enhancing the robustness of the watermark after encryption.

**Figure 2:**
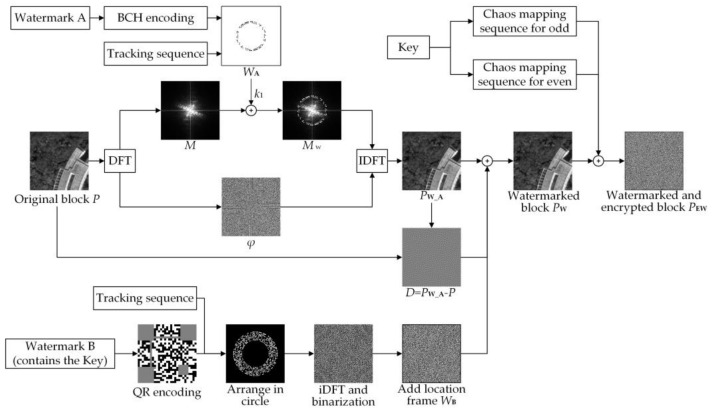
Embedding and encryption process of one block.

Figure 2 illustrates the embedding and encryption process of one block. If it is a multi-band image, we perform embedding and encryption on each band. The process can be divided into three parts. (1) Embed message A in the DFT domain. (2) Generate the watermark matrix of watermark B based on the QR code encoding method and inverse Fourier transform. Then, embed watermark B based on odd-even quantization and the difference caused by embedding message A. (3) Generate chaos mapping sequence for odd and even separately, and then perform encryption. Details are as follows:

#### 2.1.1. DFT-Based Embedding of Watermark A

Considering the possible rotation, scaling and transform (RST) attacks caused by user operations, we embed the message sequence and tracking sequence in a circle region with different radii, as shown in Figure 1 so that we can locate the tracking message through an exhaustive search after a log-polar transform of DFT coefficients, thereby resynchronizing the watermark message. Details as follows:

Step 1: We encode watermark message A by a BCH error correction code to achieve the message sequence $M_{A}=\left\{m_{A}\left(i\right)|m_{A}\left(i\right)\in \left\{0,1\right\},\;i=0,\ldots ,l-1\right\}$ and generate a 30-bit pseudorandom sequence as the tracking sequence $M_{\mathrm{TA}}=\left\{m_{\mathrm{TA}}\left(i\right)|m_{\mathrm{TA}}\left(i\right)\in \left\{0,1\right\},\;i=0,\ldots ,29\right\}$.
Step 2: Divide host image into square blocks. According to the original size of host image, set the side length of the square block to $L_{0}$. If the edge part is not enough to form blocks, it is supplemented with pixels of 0 value.
Step 3: $M_{A}$ and $M_{\mathrm{TA}}$ are embedded at $R_{1}$ and $R_{2}$ separately. The embedding coordinates of $M_{A}$ is defined as:
  (1) $$\begin{array}{c} x_{i}=\mathrm{floor}\left(\frac{L_{0}}{2}+1\right)+\mathrm{floor}\left[R_{1}\cdot \cos\left(\frac{i\cdot \pi }{l}\right)\right]\\ y_{i}=\mathrm{floor}\left(\frac{L_{0}}{2}+1\right)+\mathrm{floor}\left[R_{1}\cdot \sin\left(\frac{i\cdot \pi }{l}\right)\right] \end{array}$$
  where i is the *i*-th element of $M_{A}$. The method of calculating embedding coordinates of $M_{\mathrm{TA}}$ is the same. After this, we can achieve the watermark matrix $W_{A}\left(x_{i},y_{i}\right)$.
Step 4: Each time, input one band of one original square block P, and perform DFT transform. The watermark is embedded in the magnitude spectrum. Because the low and medium-frequency magnitude coefficients with high values can be well preserved in screen-cam process and the low values are not [44], the embedding method is defined as:
  (2) $$M_{W}\left(x,y\right)=\{\begin{array}{c} k_{1}\;,\;w\left(i\right)=1\\ \mathrm{no}\;\mathrm{change}\;,\;w\left(i\right)=0 \end{array}$$
  where $M_{W}\left(x,y\right)$ defines the watermarked magnitude spectrum and $k_{1}$ defines the embedding strength.
Step 5: Perform inverse DFT to achieve one watermarked band of one block $P_{W\_A}$. Output $P_{W\_A}$ each time.
Step 6: Repeat step 4 and step 5 to complete the embedding of all bands and blocks. Then, delete the part for supplement. The result is the watermarked image with watermark A.

#### 2.1.2. Odd-Even Quantization-Based Embedding of Watermark B

**Figure 3:**
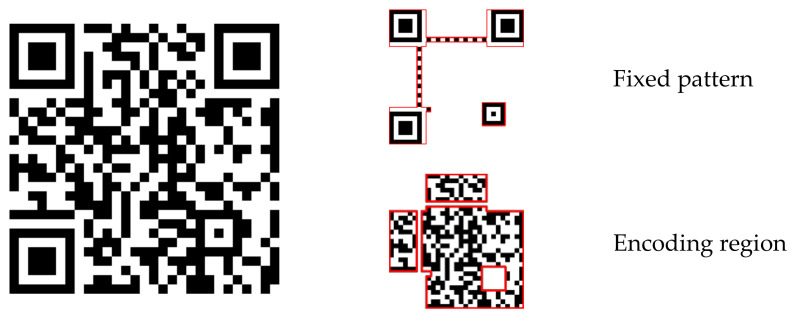
QR code architecture.

The QR code is an error correction code and has high information capacity. Therefore, it is commonly used as watermark generation method [58,59,60]. Hence, we propose a novel QR code-based watermark generation method. Details are as follows:

First, we encode watermark message sequence B by the QR code encoding method. The structure of the QR code includes the fixed pattern for resynchronization and encoding region for the message, as shown in Figure 3. Because we will rearrange the message bits, we do not need the fixed pattern. We choose the encoding region and record the encoding message line by line as $M_{B}=\left\{m_{B}\left(i\right)|m_{B}\left(i\right)\in \left\{0,1\right\},\;i=0,\ldots ,j-1\right\}$. Considering the watermark capacity, the watermark message B is designed to be not more than 42 bytes of 8 bits [61]. The message is encoded by version 3 QR code with M error correction level, and it can be recorded as a sequence of j=597 bits.

Then, as above, a 23-bit pseudorandom sequence $M_{\mathrm{TB}}=\left\{m_{\mathrm{TB}}\left(i\right)|m_{\mathrm{TB}}\left(i\right)\in \left\{0,1\right\},\;i=0,\ldots ,22\right\}$ is generated as the tracking sequence. Therefore, the whole watermark message is the combination of $M_{B}$ and $M_{\mathrm{TB}}$, a total of 620 bits.

**Figure 4:**
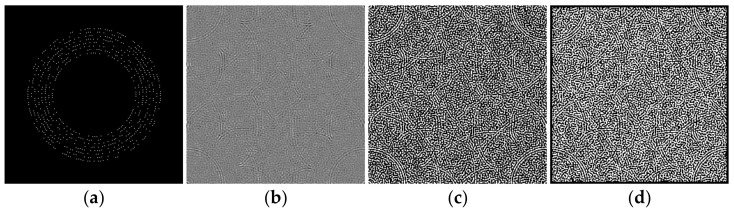
Generation process of $W_{B}$. (**a**) Arrange message sequence in circle regions of a zero matrix; (**b**) inverse Discrete Fourier transform (IDFT) of matrix (**a**); (**c**) binarization of matrix (**b**); (**d**) add a frame for matrix (**c**).

Next, we rearrange the whole watermark message sequence in circle regions of a $L_{0}\times L_{0}$ zero matrix, as shown in Figure 4a. The message bits are arranged at radii $R_{B}\left(i\right)\in \left\{60,65,70,75,80,85,90,95\right\}$. At each $R_{B}\left(i\right)$, $R_{B}\left(i\right)$ bits are arranged. For example, at radius 60, 60 bits are arranged. The coordinates are calculated based on Format (2).

After that, we perform IDFT on the matrix, as shown in Figure 4b, and binarization based on positive and negative values, as shown in Figure 4c.

Finally, we add a frame to the matrix by changing all the values within 3 from the edge to 0, as shown in Figure 4d. The result is the watermark matrix $W_{B}\left(x_{i},y_{i}\right)$.

For ensuring the imperceptibility of the proposed scheme, $W_{B}$ is hidden in the image changes caused by embedding watermark A. The proposed odd-even quantization-based embedding method only causes around 50% of the pixel values to change by 1. Therefore, it does not affect the use of watermark A.

**Figure 5:**
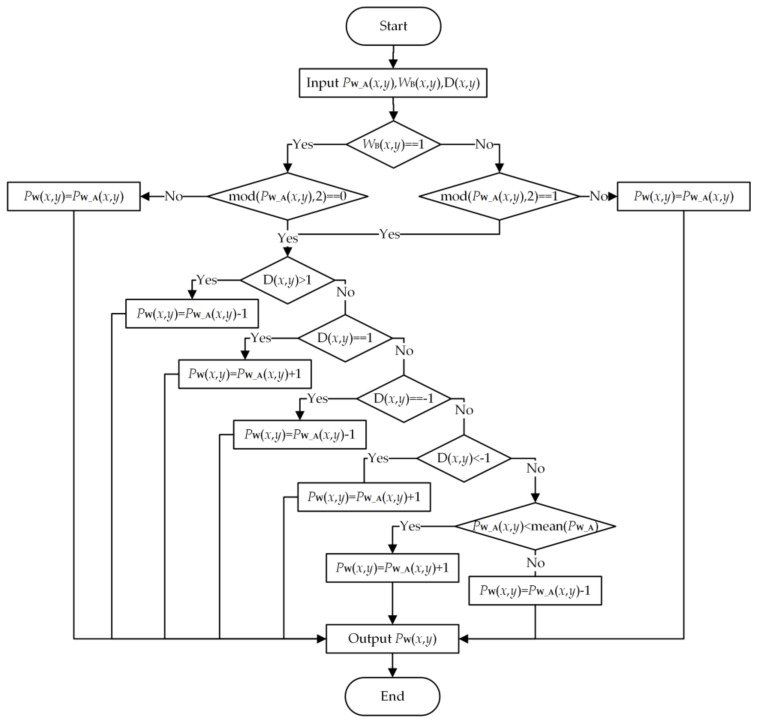
Embedding procedure of one bit of $W_{B}$.

The $W_{B}$ is also embedded block by block. The embedding process of one band of one block is as follows: First, we calculate the image changes $D=P_{W\_A}-P$ then embed $W_{B}$ bit by bit. Figure 5 illustrates the embedding procedure of one bit. The main idea is modulating the pixel values in a reverse direction of the changes caused by embedding watermark A. Finally, we achieve the watermarked band of one block $P_{W}$ with message A and message B.

#### 2.1.3. Odd-Even Segment Encryption

We encrypt odd numbers and even numbers separately into different numerical ranges based on a logistic map, which is idely used to generate a chaotic mapping sequence [1,62,63]. The logistic map is defined as:

(3) $$X_{n+1}=u\cdot X_{n}\cdot \left(1-X_{n}\right)$$

where u is the system parameter. When $X_{n}\in \left(0,1\right),\;u\in \left(3.5699456,\;4\right)$, the logistic map is chaotic.

The encryption process is as follows:

First, given the secret key, which is the combination of two initial values $X_{O}\left(0\right)$, $X_{E}\left(0\right)$ and a parameter u two one-dimensional array $V_{1}\left(i\right)$ and $V_{2}\left(i\right)$ with a length of $L_{0}$ are generated by iterating $2\cdot L_{0}$ times through Equation (3), respectively. $L_{0}$ depends on the data type of the image, where $L_{0}=2^{bit\;depth-1}$.

Then, sort $V_{1}\left(i\right)$ from the smallest to largest to obtain array $V_{s1}\left(i\right)$, and record the index that elements of $V_{s1}\left(i\right)$ in $V_{1}\left(i\right)$ as $V_{O}\left(i\right)$. For example, suppose element $V_{1}\left(9\right)$ becomes element $V_{s1}\left(0\right)$ after sorting, then $V_{O}\left(0\right)=9$. Perform the same process on array $V_{2}\left(i\right)$ to obtain $V_{E}\left(i\right)$. $V_{O}\left(i\right)$ and $V_{E}\left(i\right)$ with a length of $L_{0}$ are the two chaos mapping sequences for odd and even values separately.

**Figure 6:**
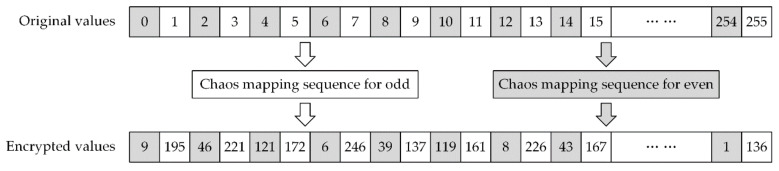
Example of pixel encryption.

Finally, the encryption method is defined as:

(4) $$\begin{array}{ll} P_{\mathrm{EW}}\left(x,y\right)=V_{E}\left(P_{W}\left(x,y\right)/2\right) & if\;P_{W}\left(x,y\right)\;is\;even\\ P_{\mathrm{EW}}\left(x,y\right)=V_{O}\left((P_{W}\left(x,y\right)-1)/2\right)+L_{0} & if\;P_{W}\left(x,y\right)\;is\;odd \end{array}$$

where $P_{\mathrm{EW}}$ defines the encrypted and watermarked image. An example of pixel encryption is shown in Figure 6, where all original even values are encrypted to low values and original odd values are encrypted to high values.

**Figure 7:**
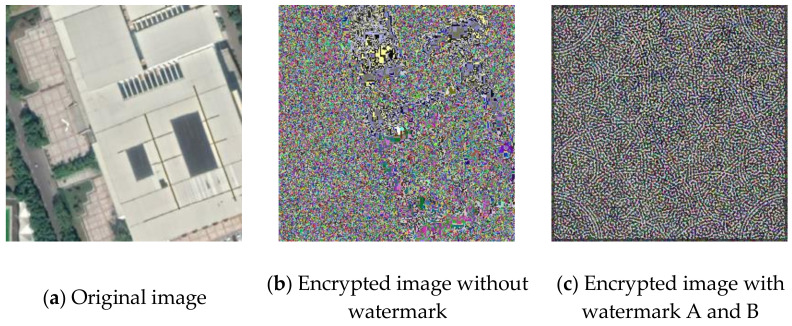
Example of Encryption Result.

The weakness of directly modifying the pixel values based on a mapping sequence for encryption is that the shape of the area with the same pixel values can still be seen after the encryption, as shown in Figure 7a,b. Fortunately, after watermark embedding, most of the same pixel values in one area will become different, which means it will effectively avoid the weakness, as shown in Figure 7c.

### 2.2. Extraction and Decryption Scheme

**Figure 8:**
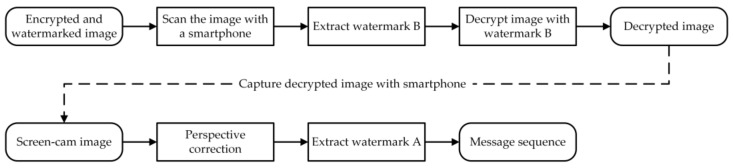
Framework of extraction and decryption process.

Figure 8 shows the extraction and decryption process. Nowadays, authentication and secret key management through smartphones are already mature technologies. When receiving the encrypted and watermarked image, authorized users can use smartphones to detect and extract watermark B by canning or photographing with a proprietary application. Then, the secret key and other information are obtained by decoding watermark B to decrypt the image.

If the decrypted image is photographed without authorization, watermark A can be extracted from the screen-cam image to hold data leakage accountability. Because watermark A is designed for leakage tracking, manual operation is acceptable. For screen-cam images, we perform perspective correction of the recaptured image and crop out the needed part for watermark A extraction. This part is divided into blocks, and watermark A is detected block by block. Next, we locate the tracking sequence by calculating the cross-correlation to estimate the positions of the embedded bits. Finally, message A is extracted and decoded.

#### 2.2.1. Watermark B Extraction and Decryption

**Figure 9:**
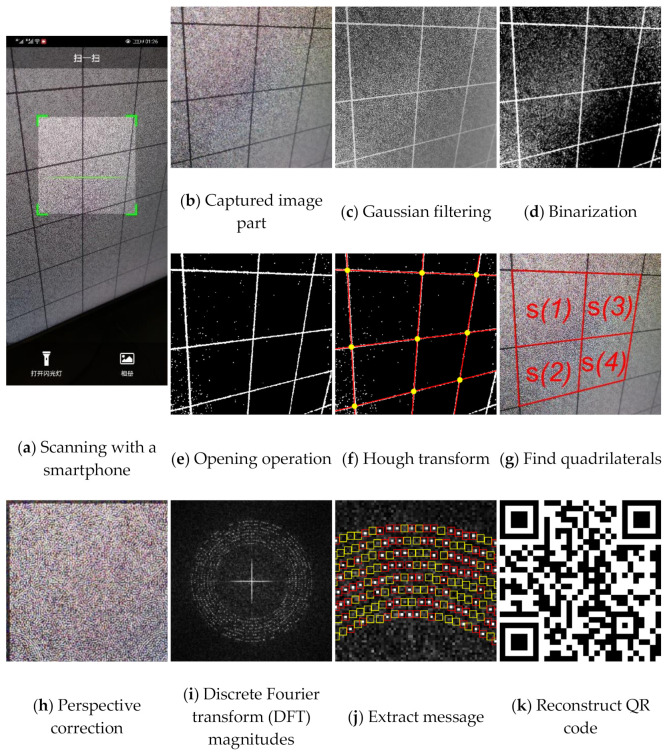
Detection and extraction process of watermark B.

Using smartphones to extract watermark B from the encrypted image is like using smartphones to read QR code. Because today’s smartphones have high-megapixel cameras, the captured image will be highly zoomed-in compared with the original image displayed on the screen when shooting at a close distance. Therefore, for a screen-cam image scanned, as shown in Figure 9a, we zoom and crop out the needed part first. According to the camera resolution of the smartphone, we crop and zoom out the captured image accordingly to obtain image $I_{b}$, as shown in Figure 9b. The perspective correction, message extraction and decryption process are as follows:

1. Perspective correction:
  Step 1: Input $I_{b}$. Convert $I_{b}$ to grayscale $I_{g}$ and calculate $I_{g}^{\prime }=255-I_{g}$. Then, perform Gaussian filtering with a two-dimensional Gaussian kernel $H_{1}$, where sigma is set to 1 and window size is set to 6. Hence, the $I_{c}\left(x,y\right)=H_{1}*I_{g}^{\prime }\left(x,y\right)$ is obtained by a convolution process, as shown in Figure 9c.
  Step 2: Binarize $I_{c}$ based on a threshold $T_{1}$ to obtain binary image $I_{d}$, as shown in Figure 9d. Then, perform opening operation, which is erosion and dilation in turn, with structuring element se to obtain $I_{e}$, as shown in Figure 9e.
  Step 3: Perform Hough transform to search the lines from $I_{e}$, and calculate the intersection points of these lines within the image range, as shown in Figure 9f. Record the coordinates of these points as $p_{i}$.
  Step 4: Perspective transformation needs four pairs of points [43]. The side length $L_{0}$ is known, which means we know transformed coordinates of the four corners of one block. Therefore, we can select four corner points of one block for perspective correction. Select and construct $p_{i}$ into a point set $S=\left\{s\left(i\right)|s\left(i\right)\in \left\{p_{1},p_{2},p_{3},p_{4}\right\},i=0,\ldots ,n\right\}$, which contains all candidate point sets that can be used for perspective correction, based on the searching method in [44]. The s(i) are sorted according to the sum of the distances between the points from largest to smallest. An example of the quadrilaterals formed by each s(i) is shown in Figure 9g. The s(1) is selected for message extraction.
  Step 5: The perspective correction process is defined as:
    (5) $$\left[\begin{array}{c} x\prime \\ y\prime \\ 1 \end{array}\right]=H_{2}\left[\begin{array}{c} x\\ y\\ 1 \end{array}\right],\;\mathrm{where}\;H_{2}=\left[\begin{array}{ccc} a_{11} & a_{12} & a_{13}\\ a_{21} & a_{22} & a_{23}\\ a_{31} & a_{32} & a_{33} \end{array}\right]$$
    where ${\left[x^{\prime },y^{\prime },1\right]}^{T}$ and ${\left[x,y,1\right]}^{T}$ define the homogeneous point coordinates of the corrected image and the captured image, respectively. $H_{2}$ is a nonsingular 3 × 3 homogeneous matrix. By using $s\left(1\right)\in \left\{p_{1},p_{2},p_{3},p_{4}\right\}$, and setting the four corresponding points representing the four corners of a corrected block as $\left\{p_{1}^{\prime },p_{2}^{\prime },p_{3}^{\prime },p_{4}^{\prime }\right\}$, the $H_{2}$ can be calculated. Then, we perform perspective correction of $I_{b}$ and crop out the block $I_{h}$, as shown in Figure 9h. Output $I_{h}$.
2. Message extraction:

We use the grayscale of $I_{h}$ and perform DFT to obtain the magnitude spectrum $I_{i}$, as shown in Figure 9i. The encrypted image is a noise image, which means the image itself does not have high magnitude values around the embedding region. In other words, the modulated high magnitudes for message embedding are significant.

Furthermore, the manually perspective correction cannot be perfect, which means it will cause the shifting of magnitude coefficients [44]. Therefore, we use the maximum value $v_{m}\left(i\right)$ within a 3 × 3 region centered at the embedding coordinates to determine the message bit $w_{B}^{\prime }\left(i\right)$, as shown in Figure 9j, where red boxes and yellow boxes are the 3 × 3 areas of the positions where the embedded message bit is ‘1’ and ‘0’, respectively. The extraction method of watermark B is defined as:

(6) $$w_{B}^{\prime }\left(i\right)=\{\begin{array}{l} 1,\;if\;v_{m}\left(i\right)>T_{B}\\ 0,\;otherwise \end{array}$$

(7) $$T_{B}={\bar{E}}_{B}+k_{2}\sigma_{B}$$

where $T_{B}$ is the set threshold, ${\bar{E}}_{B}$ and $\sigma_{B}$ are the mean value and the standard deviation of all the magnitudes in the range of [60,95], $k_{2}$ is a fixed value.

Although $I_{h}$ is corrected to the original size, because it is square we still need to consider whether the image is under a rotation by 90°. Based on the nature of Fourier domain, we can easily calculate the coordinates of the embedded tracking sequence $M_{\mathrm{TB}}$ in these two cases. Therefore, based on Equations (6) and (7), we extract the messages $M_{\mathrm{TB}}^{\prime }\left(1\right)$ and $M_{\mathrm{TB}}^{\prime }\left(2\right)$ from the positions of embedded tracking sequence in both cases. If the erroneous bits in either of $M_{\mathrm{TB}}^{\prime }\left(1\right)$ and $M_{\mathrm{TB}}^{\prime }\left(2\right)$ are less than the given threshold $T_{2}$, we consider the watermark exists.

$M_{B}^{\prime }$ is then extracted also based on Equations (6) and (7). Based on the inverse process of the watermark generation method, the QR code is reconstructed with $M_{B}^{\prime }$, as shown in Figure 9k. Finally, by decoding the QR code, the watermark message B containing the decryption key is obtained.

3. Decryption:

Based on the extracted decryption key and the bit depth of the image, the same $V_{O}\left(i\right)$ and $V_{E}\left(i\right)$ can be calculated. The decryption process is defined as:

(8) $$P_{W}^{\prime }\left(x,y\right)=\{\begin{array}{ll} f_{index}\left(V_{E},P_{\mathrm{EW}}\left(x,y\right)\right)\cdot 2 & ,\;if\;P_{\mathrm{EW}}\left(x,y\right)<2^{bit\;depth-1}\\ f_{index}\left(V_{O},P_{\mathrm{EW}}\left(x,y\right)\right)\cdot 2+1 & ,\;else \end{array}$$

where $P_{W}^{\prime }$ defines the decrypted and watermarked image and function $f_{index}\left(x,y\right)$ defines returning the index of element x(i) that equals to y. bit depth defines the bit depth refers to the image format.

#### 2.2.2. Watermark A Extraction

**Figure 10:**
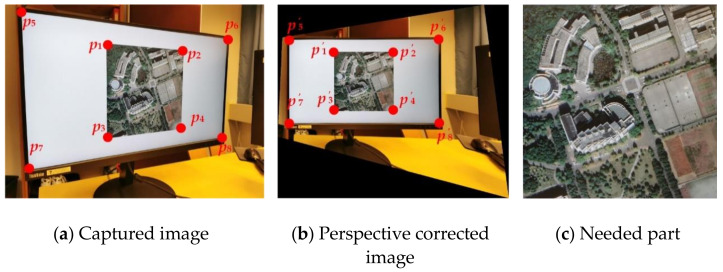
Perspective correction process.

For a screen-cam image, we perform perspective correction by manually selecting four points. As watermark A is designed for leakage tracking, manual selection is acceptable. As shown in Figure 10, we can use the four corner points of the host image $\left\{p_{1},p_{2},p_{3},p_{4}\right\}$ or the four corner points $\left\{p_{5},p_{6},p_{7},p_{8}\right\}$ of the screen to correct the captured image to the original size. Then, the portion needed for watermark detection is cropped. If the original size of the image and the screen are unknown, because the watermark A is robust to scaling attack, we can also correct the captured image to an image with the original aspect ratio. As we mentioned in Section 2.2.1, a slight accuracy error in corner point selection and resulting shift of magnitude coefficients is acceptable, because we perform watermark extraction based on the maximum value within 3 × 3 region of the embedded watermark position.

**Figure 11:**
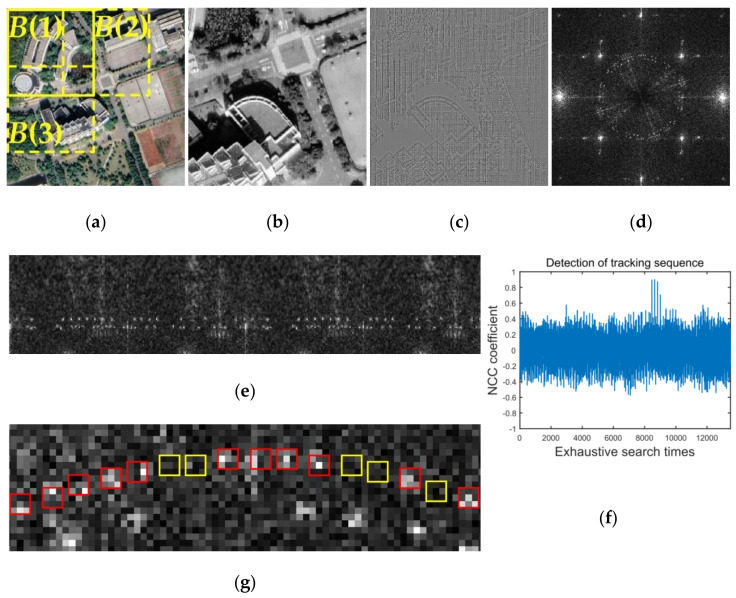
Detection and extraction process of watermark A. (**a**) Examples of blocks can be used for watermark detection; (**b**) luminance spectrum of one block; (**c**) noise component of one block; (**d**) DFT domain of noise component; (**e**) map detection range from Cartesian coordinates to polar coordinates; (**f**) calculate NCC coefficients; (**g**) 3 × 3 region centered at embedding coordinates.

According to the nature of the DFT domain, the message embedded in the magnitude spectrum is distributed in the whole image. The message embedded in each block is the same. Therefore, any part of the image can be used for watermark detection and extraction. Considering the severe distortion caused by a screen-cam attack, we can use a square block B(i) with a side length of $L_{1}$, which is larger than $L_{0}$, for detection. Furthermore, if there is no watermark, the DFT magnitude coefficients of the blocks with a small amount of overlap are very different, which will not cause a false alarm. Therefore, the blocks used for detection do not need to be completely nonoverlapping. As shown in Figure 11a, the overlapping block B(1), B(2), and B(3) can all be used for watermark detection at the same time. Therefore, we choose the blocks in turn with a step of $0.7\cdot L_{1}$ at both horizontal and vertical directions.

Because the size of a selected block B(i) is larger than an embedded block, the positions of embedded messages are changed accordingly. Besides, watermarks can be considered as a form of noise [64]. Detecting the watermark from the noise component can reduce the negative impact of the image itself [44]. Therefore, to resynchronize the watermark, we locate the embedded tracking sequence from the noise component using the normalized cross-correlation (NCC) function. The noise component $B_{n}$ is defined as:

(9) $$B_{n}=B_{l}-H_{3}*B_{l}$$

where $B_{l}$ defines the luminance spectrum of selected block B and $H_{3}$ defines a 3 × 3 spatial domain Wiener filter. Figure 11b,c show examples of a $B_{l}\left(i\right)$ and $B_{n}\left(i\right)$. 

We transform $B_{n}$ to the DFT domain $B_{f}$, as shown in Figure 11d. Considering the size and scaling difference between B(i) and the original block, the detection range is set from radius 50 to radius 150. Mapping the detection range from Cartesian coordinates to polar coordinates is done, as shown in Figure 11e. Then, we perform an exhaustive search by calculating the NCC coefficients between the extracted coefficients and the tracking sequence $M_{\mathrm{TA}}$, which is defined as:

(10) $$C\left(j\right)=\frac{\sum_{i=0}^{29}\left(V_{\mathrm{TA},\;j}^{\prime }\left(i\right)-\bar{V_{\mathrm{TA},\;j}^{\prime }}\right)\left(M_{\mathrm{TA}}\left(i\right)-\bar{M_{\mathrm{TA}}}\right)}{\sqrt{\sum_{i=0}^{29}\left(V_{\mathrm{TA},\;j}^{\prime }\left(i\right)-\bar{V_{\mathrm{TA},\;j}^{\prime }}\right)\sum_{i=0}^{29}\left(M_{\mathrm{TA}}\left(i\right)-\bar{M_{\mathrm{TA}}}\right)}}$$

where C(j) defines the NCC coefficient of *j*-th search and $V_{\mathrm{TA},\;j}^{\prime }$ defines the extracted message sequence of the *j*-th search. $\bar{V_{\mathrm{TA},\;j}^{\prime }}$ and $\bar{M_{\mathrm{TA}}}$ defines the mean of extracted message sequence and the original tracking sequence. $V_{\mathrm{TA},\;j}^{\prime }\left(i\right)$ is the maximum coefficient value within the 3 × 3 region centered at the detection position. Because of this, if the watermark exists, more than one high NCC coefficient may be calculated. An example of calculation resulting from Figure 11e is shown in Figure 11f. If the maximum value of C(j) is greater than 0.65, which is an experimental threshold, we consider the positions of corresponding $V_{\mathrm{TA},\;j}^{\prime }$ is the positions of embedded tracking sequence.

Based on the detected tracking sequence, we can estimate the positions and the radius $R_{1}^{\prime }$ of embedded $M_{A}$ in $B_{f}$. Because the polar mapping process interpolates the data, which causes a slight change, we extract the watermark message from $B_{f}$ directly. The extraction method of watermark A is the same as watermark B, the maximum value $v_{m}\left(i\right)$ within the 3 × 3 region centered at the embedding coordinates is used to determine the message bit $w_{A}^{\prime }\left(i\right)$, as shown in Figure 11g, but with different parameters. The extraction method of watermark A is defined as:

(11) $$w_{A}^{\prime }\left(i\right)=\{\begin{array}{l} 1,\;if\;v_{m}\left(i\right)>T_{A}\\ 0,\;otherwise \end{array}$$

(12) $$T_{A}={\bar{E}}_{A}+k_{3}\sigma_{A}$$

where $T_{A}$ is the set threshold, ${\bar{E}}_{A}$ and $\sigma_{A}$ are the mean value and the standard deviation of all the magnitudes in the range of $\left[R_{1}^{\prime }-2,R_{1}^{\prime }+2\right]$ and $k_{3}$ is a fixed value. Finally, an extracted watermark message $W_{A}^{\prime }\left(i\right)$ is obtained by BCH decoding. To avoid a false positive, watermark detection is successful only when two of $W_{A}^{\prime }\left(i\right)$ are the same. The same decoded message will be used as extraction result.

## 3. Experimental Results

In our experiment, we set message A to 24 bits, which means it can support 16,777,216 IDs. The ID sequence was encoded by BCH (63.24) to generate $M_{A}$ with 63 bits, which can correct 7 error bits. Watermark B was set as {key = 8190/1713/398232; level = NNU; ID = 15821018}, including the decryption key, user ID, and other information.

To ensure the size of a block for resynchronizing from an encrypted image is applicable for practical application, $L_{0}$ was set to 256. The middle frequency coefficients at $R_{1}=60$ and $R_{2}=55$ were selected to embed $M_{A}$ and $M_{\mathrm{TA}}$, respectively. The threshold $T_{2}$ was set to 4. Because $M_{\mathrm{TB}}$ is 23 bits, the false positive rate for judging whether watermark B exists can be calculated as $\overset{23}{\underset{23-T_{2}+1}{\sum }}{\left(0.5\right)}^{23}\cdot \left(\frac{23!}{3!20!}\right)$=2.44E-04 [65]. This false positive rate is not very low. Fortunately, if the reconstructed QR code based on extracted watermark B is wrong, it cannot be decoded. This can also be regarded as double insurance to prevent false positives.

The monitor we used was a 27-inch ‘ThinkVision P27q’ monitor with 2560 × 1440 pixels. The photography equipment we used was a P30PRO smartphone with a 40 MP pixel camera. The application for extracting the watermark from encrypted images was developed by Java running on the platform of P30PRO. The rest of the experiments were performed by Matlab 2019b on a Windows 10 operation system with an Intel i7-9700 CPU. The host data was five images from database [66] and five images restitched by tile images obtained from Google Earth.

In Section 3.1, the selection of parameters through statistical experiments is presented. In Section 3.2, the security of encryption scheme is discussed. In Section 3.3, the robustness of the watermark B against screen-cam attack is analyzed. In Section 3.4 and Section 3.5, we verify the robustness of watermark A against common image processing attacks and screen-cam attacks, respectively. As our method can achieve partial decryption, in Section 3.6, we verify the robustness of the partially decrypted image against screen-cam attack.

### 3.1. Parameter Settings

#### 3.1.1. Selection of Embedding Strength $k_{1}$

**Figure 12:**
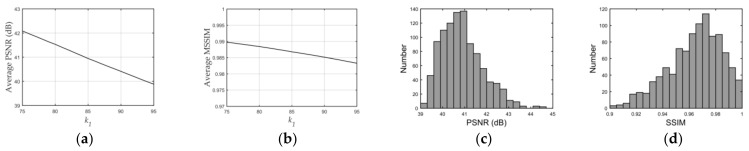
PSNR and SSIM with different and selected $k_{1}$. (**a**) and (**b**) are average PSNR and SSIM values with different $k_{1}$; (**c**) and (**d**) are PSNR and SSIM values with selected $k_{1}$.

**Figure 13:**
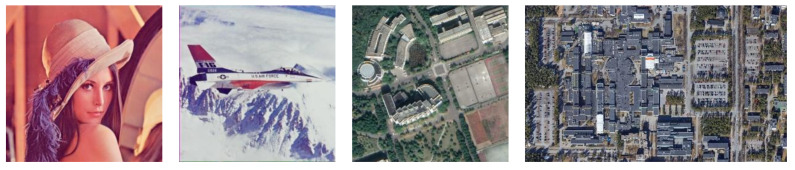

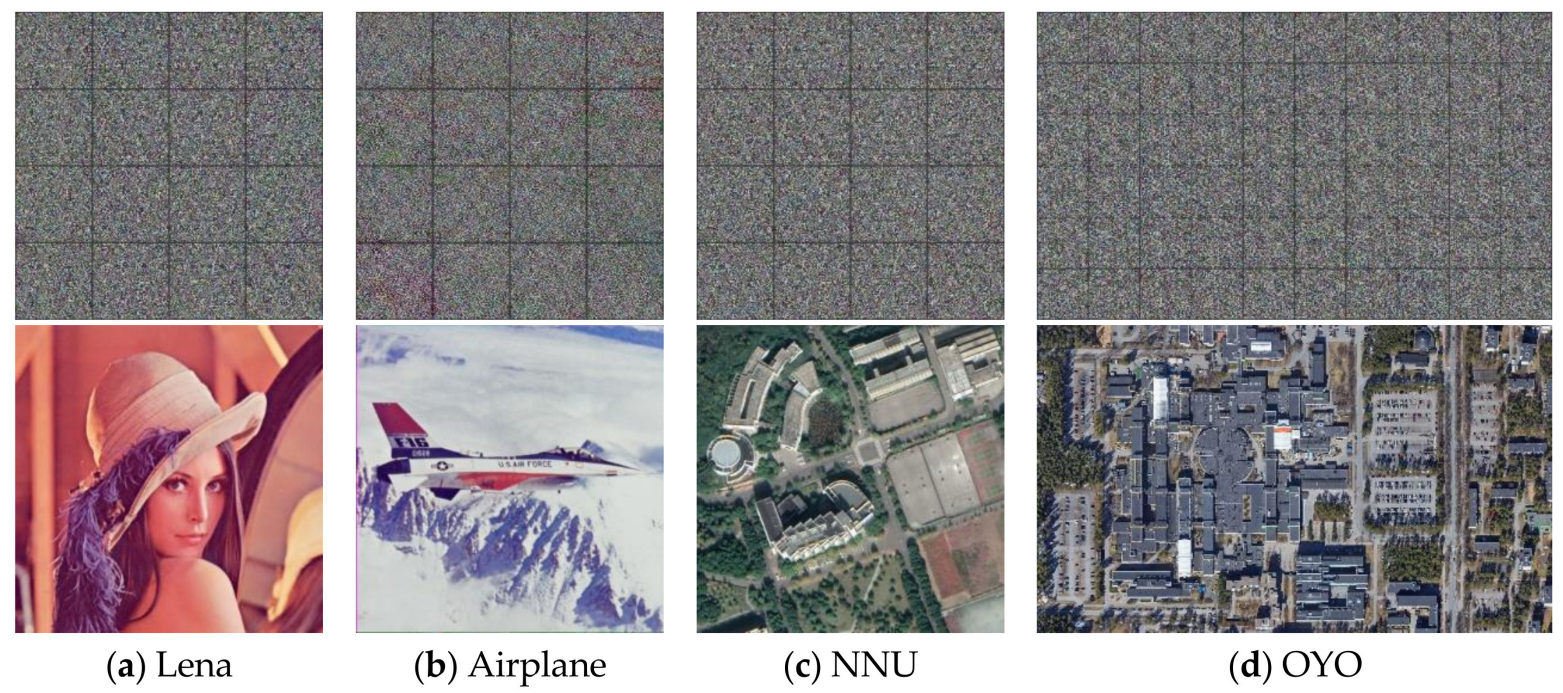
Examples of host images (**first row**), encrypted and watermarked images (**second row**), and decrypted and watermarked images (**third row**).

Embedding strength balances the robustness and imperceptibility of the proposed scheme. One thousand tile images obtained from Google Earth were utilized for statistical experiments to select the appropriate embedding strength $k_{1}$ in Equation (1). Image quality degradation was evaluated by the widely used peak signal-to-noise ratio (PSNR) and structural similarity index (SSIM) [67]. The average PSNR and SSIM values of the embedded images with different $k_{1}$ are shown in Figure 12a,b. In order to ensure the PSNR values of most images after embedding was greater than 40, we set $k_{1}$ to 85. The average PSNR was 40.9446 dB and the average SSIM was 0.9868. With the selected $k_{1}$, the PSNR and SSIM values of all the test images after embedding are shown in Figure 12c,d. The examples of original the host image, encrypted and watermarked host images and decrypted and watermarked host images are shown in Figure 13.

#### 3.1.2. Selection of Threshold $T_{1}$ and Structuring Element se for Synchronization

To ensure the success of the automatic perspective correction of recaptured encrypted images, we need to select the most suitable threshold $T_{1}$ for binarizing and structuring element se. According to the shooting distance, the camera resolution and the screen resolution, the scaling ratio of the captured images is quite different. To process the recaptured images of different scaling levels, the required parameters vary greatly. Therefore, we tested the performance of different parameters with different shooting distances.

When we use a smartphone to scan the code on the screen, the distance between the smartphone and the screen is commonly within 40 cm. Therefore, we counted the results of automatic perspective correction with different $T_{1}$ and se at the shooting distance of 10 cm, 20 cm, 30 cm and 40 cm, and the shooting angle of 0 degree perpendicular to the screen. Because our photography equipment had a high resolution that causes the captured image to be zoomed, we set the captured image to zoom 60% before the processing. The structuring element se we used here consisted of only ‘1′.

The results are shown in Table 1, Table 2 and Table 3, where ‘√’ defines perspective correction succeeded and ‘×’ defines it failed. As shown in Table 1, Table 2 and Table 3, there are three groups of $T_{1}$ and se that can satisfy all the scenarios in our experiment. Therefore, we chose one of the three groups. In our experiment, we set $T_{1}=0.65$ and se with 5 × 5 size.

#### 3.1.3. Selection of Side Length $L_{1}$ of Detection Block for Watermark A Extraction

**Figure 14:**
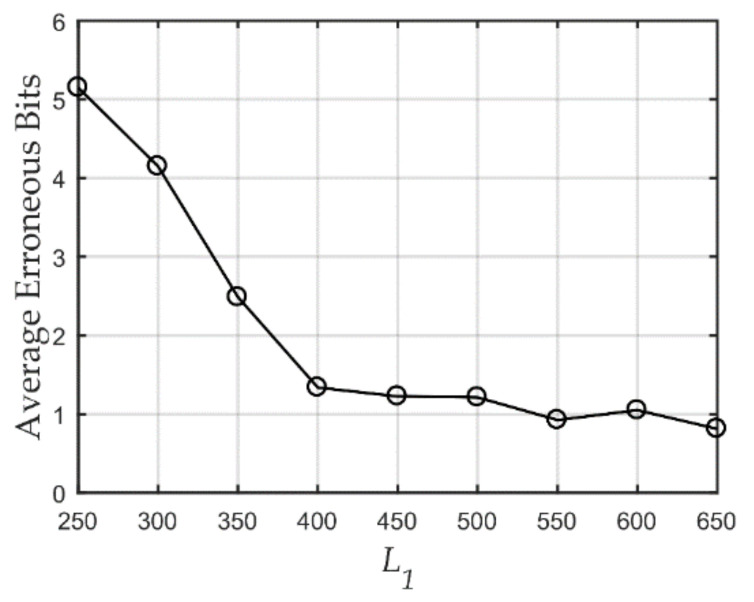
Average erroneous bits with different $L_{1}$.

In theory, the larger the size of selected block B(i) in watermark detection, the clearer the watermark information should be. However, considering the size of the original image is restricted, and the image needs to be divided into multiple blocks for watermark detection, the size of B(i) should be as small as possible. To balance this contradiction, we analyzed the number of erroneous bits when using B(i) with different side length $L_{1}$ in the watermark extraction. In this experiment, we set the shooting distance from 30 cm to 100 cm at an interval of 10 cm and the shooting angle to 0 degrees. Hence, 80 captured images of the 10 host images were utilized. The average erroneous bits with different $L_{1}$ are shown in Figure 14. When the $L_{1}$ was greater than 400, although the number of erroneous bits was lower, the variation tended to be stable. Therefore, to ensure low erroneous bits and also low side length, we set $L_{1}=400$.

#### 3.1.4. Selection of the Fixed Value $k_{2}$ and $k_{3}$ for Message Extraction Threshold

**Figure 15:**
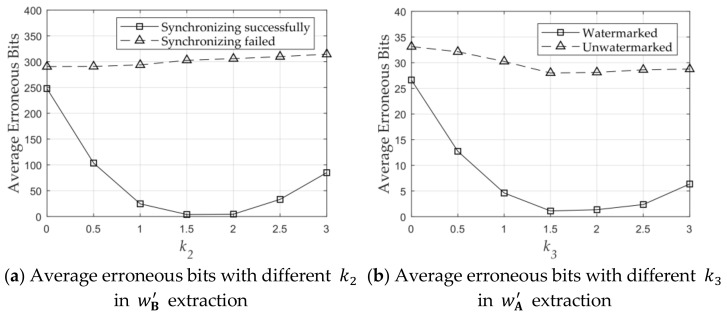
Erroneous bits with different message extraction thresholds.

According to Equations (6), (7), (11), and (12), the fixed value $k_{2}$ and $k_{3}$ are used to calculate the detection threshold for $w_{B}^{\prime }$ and $w_{A}^{\prime }$, respectively, which can determine the validity of the message extraction result. Based on the 80 captured images mentioned in Section 3.1.3, we analyzed the number of erroneous bits with different thresholds. We analyzed the extracted result of synchronizing failed and unwatermarked. The number of average erroneous bits when synchronizing failed or unwatermarked was independent of threshold, is shown in Figure 15. When $k_{2}=1.5$, as shown in Figure 15a, and $k_{3}=1.5$, as shown in Figure 15b, we achieved the minimum average erroneous bits in extracting $w_{B}^{\prime }$ and $w_{A}^{\prime }$, respectively. Therefore, $k_{2}$ and $k_{3}$ were both set to 1.5.

### 3.2. Security of Encryption

**Figure 16:**
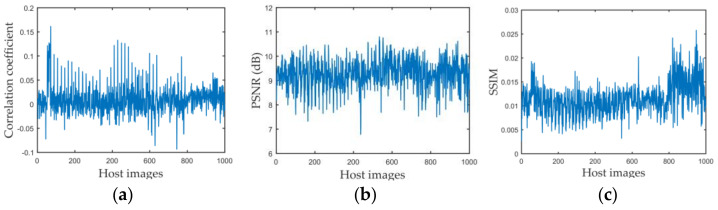
Evaluation of proposed encryption method. (**a**–**c**) are correlation coefficients, PSNR and SSIM values, respectively.

We used three commonly used statistical analysis metrics [68,69] to measure encryption security. The experiment data was the 1000 images we obtained from Google Earth. First, we performed a correlation analysis. Because two adjacent pixels in a plain image are strongly correlated vertically and horizontally [3], a good encryption method needs to reduce this correlation, which means the correlation coefficient should be near to 0. The correlation coefficient between the encrypted image and decrypted image of the watermarked image is shown in Figure 16a, where the a-axis means the serial number of host images. The average correlation coefficient of the test images was 0.0091.

Then, the PSNR and SSIM were used to analyze image degeneration and similarity between the encrypted and decrypted images. The results are shown in Figure 16b,c. The average PSNR value was 9.2738 dB, and the average SSIM was 0.0113.

**Figure 17:**
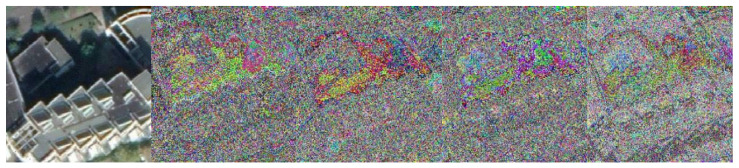

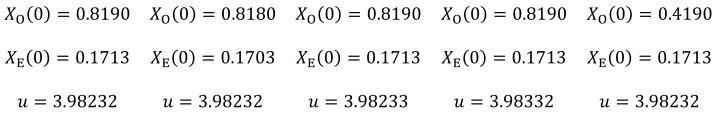
Decryption results with different key.

An ideal encryption scheme should be sensitive to the secret key, which means if a single bit in the original key is modified, the image remains unrecoverable. As our secret key was the combination of $X_{O}\left(0\right)$, $X_{E}\left(0\right)$, and u, we used different parameters to decrypt the image. An example is shown in Figure 17, where the first row is the decryption results, and the second row is the corresponding secret key used. The first image in Figure 17 is decrypted with the right key. Decryption with the wrong key cannot be recovered, even when the difference to original secret key is minimal.

### 3.3. Robustness of Watermark in Encrypted Image against Screen-Cam Attack

This section verifies the robustness of watermark B in encrypted images against screen-cam attack with different shooting conditions. Considering the real use requirements, using a smartphone to read the watermark from an encrypted image in real time is similar to using a smartphone to scan a QR code, where the phone is usually close to the screen. Therefore, in our experiment, we set the shooting distance at {10 cm, 20 cm, 30 cm, 40 cm} and the shooting angle at {0°, 15°, 30°, 45°} of horizontal left.

We employed the commonly used metrics Bit Error Rate (BER) to measure robustness. BER is defined as the ratio of the number of erroneous bits to the length of the message sequence.

Table 4 lists the average BER in extracting the watermark from encrypted images with different shooting conditions. Table 5 shows a set of examples when the shooting angle was 45°. The encrypted images are not related to the original images, and all encrypted images are similar to the noise images. Therefore, the image itself does not have high magnitude coefficients at the embedding region of the DFT domain, which makes the coefficients of embedded watermark bit ‘1’ significantly different from other coefficients. Hence, the BER can be maintained very low. The proposed method has high robustness to this situation.

When shooting at a long distance, the captured image may contain more interference factors, which will affect the automatic perspective correction. Table 6 lists some examples. These captured images cannot be automatically corrected with the proposed automatic perspective correction method. However, after simple cropping and scaling, the watermark can be effectively detected and extracted, as shown with the experiments. In practice, in real applications, we can design a zoom and partial cropping function for the watermark reading application to achieve watermark extraction at a long shooting distance.

### 3.4. Robustness of Watermark in Decrypted Image Against Common Attacks

The proposed scheme is aimed at screen-cam attacks but, at the same time, it can resist common image processing attacks. In this section, we verify the robustness of watermark A in a decrypted image to common attacks and compare the proposed scheme with three existing schemes, which are all mainly designed for print-cam or screen-cam attacks. For fair comparison, we adjusted the parameters and the size of embedding blocks of the three algorithms accordingly. The block size was set to 256 × 256 in [32], and embedded message was 64 bits. The embedding unit of one bit was changed from 8 × 8 to 16 × 16 in [43], and the embedded message was 63 bits. The method of [44] embeds 93 bits. In comparison, we set the watermarking imperceptibility of these methods at the same level by adjusting the embedding strength to keep the PSNR values similar. An example is shown in Table 7.

Table 8 lists the average BER of host images under different common image processing attacks, where—defines not robust to this attack. As shown in Table 8, the proposed scheme had better performance against most common image processing attacks.

The proposed method had high robustness to JPEG compression, where the message can still be recovered correctly under JPEG compression with QF = 20. With regard to scaling attack, we extracted the watermark message without correcting the image to its original scale. Method [32] was not robust to large scaling distortion. Method [43] needed to correct the image to original size, which was also not robust. The proposed method had better robustness than method [44] to scaling distortion. When scaling to 50%, only the proposed method could extract the watermark message completely. Rotation and cropping attack in Table 8 means the rotated image was cropped to the original size. Method [32,43] could not detect this kind of desynchronization. The synchronization method of [44] had limitations on the angle of rotation. The proposed method could resist any angle of rotation attack. With regard to median filter attack, although method [43] had the best performance, our method also performed well in comparison. Furthermore, the proposed scheme had good robustness to different types of noise attack and image enhancement process, and lower BER than the other three methods.

### 3.5. Robustness of Watermark in Decrypted Image against Screen-Cam Attack

**Figure 18:**
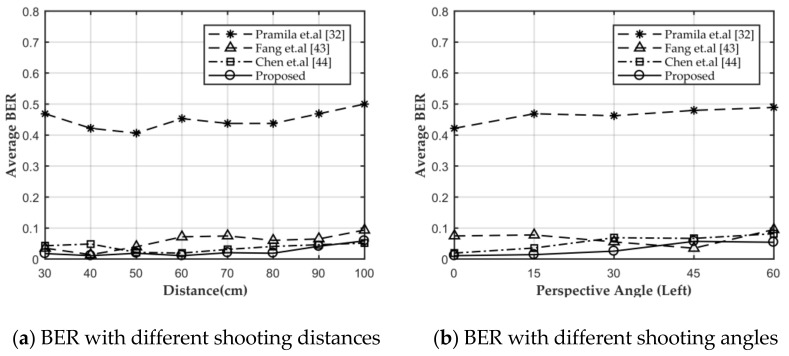
Comparison of different methods with different shooting conditions.

In this section, the robustness of watermark A in the decrypted image against screen-cam attack is tested. First, we performed a comparison with the three methods mentioned above with different shooting distances and shooting angles. Because method [44] was designed for automatic perspective correction, for fair comparison, we manually corrected the captured image if the automatic correction algorithm did not work. When shooting direction was perpendicular to the screen, the average BER of all methods with different shooting distances is shown in Figure 18a. When shooting at a distance of 60 cm, the average BER of all methods with shooting angle from perpendicular to 60° of horizontal left is shown in Figure 18b. The proposed method and method [43,44] had similar robustness against screen-cam attack.

**Figure 19:**
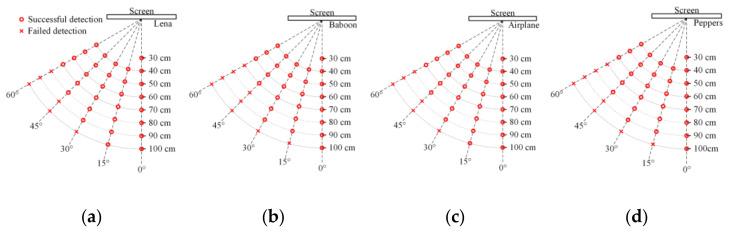

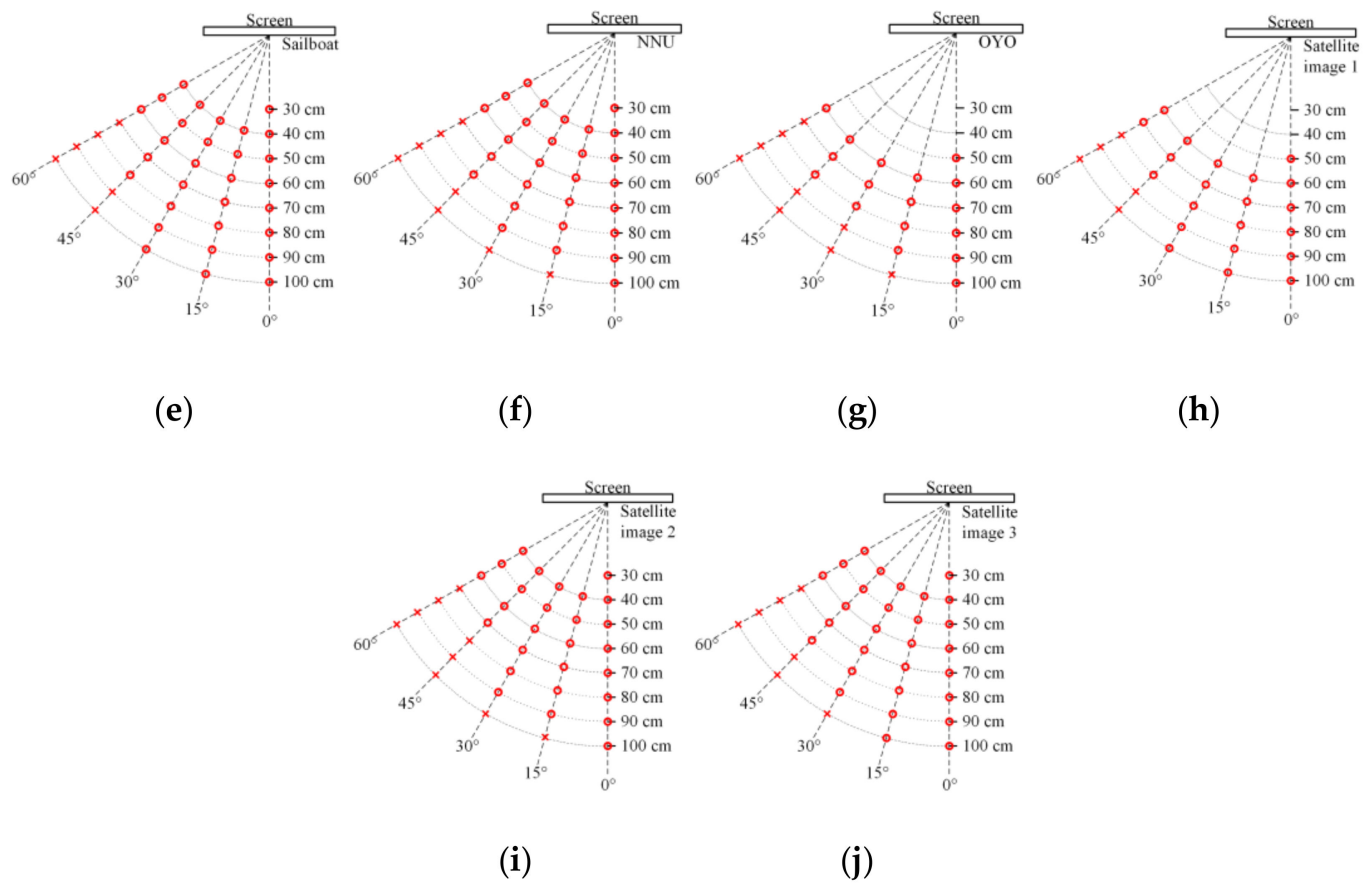
Watermark detection results against screen-cam attack. (**a**–**j**) are the detection result of Lena, Baboon, Airplane, Peppers, Sailboat, NNU, OYO, Satellite image 1, Satellite image 2, and Satellite image 3, respectively.

We verified the robustness of the proposed scheme with more shooting conditions. Because, in theory, the distortions caused by shooting at the same angle of horizontal perspective or vertical perspective are similar, only the distorted part in the host image is different. Therefore, in this experiment, we set the shooting from being perpendicular to the screen up to 60° of horizontal left at intervals of 15°. Besides, the shooting distance was set from 30 cm to 100 cm at intervals of 10 cm. When shooting with an angle to capture the whole image, the closest shooting distance was adjusted to 40 cm. Experimental results are shown in Table 9, where the average BER did not include the case where the tracking sequence was not detected, and ‘/’ defines the tracking sequence is not detected in all captured images. Figure 19 shows the watermark detection result of different host images. Table 10 lists the recovered image NNU from captured images with different shooting conditions and the corresponding BER.

Because OYO and satellite image 1 are big size images, to capture the whole host image, the closest shooting distance was adjusted as shown in Figure 19g,h. When shooting perpendicular to the screen, the watermark could be extracted at all shooting distances with low BERs. When the shooting angles were 15° and 30°, the watermark could be extracted basically at a shooting distance below 90 cm, also with low BERs. When the shooting angle was 45°, the watermark could be extracted from most captured images taken within 80cm. When shooting at a large angle of 60°, the watermark could still survive at a close shooting distance.

The captured images in the experiment above were obtained with the help of a tripod. In a real scene, we captured the images by holding a smartphone, which causes camera shake and leads to more blurring. Therefore, we also test the performance with handheld shooting. The results of some cases are shown in Table 11, showing good performance.

### 3.6. Robustness of Watermark in Partial Decrypted Image Against Screen-Cam Attack

For a screen-cam partially decrypted image, we can extract the watermark information from both the encrypted part or the decrypted part. Therefore, in essence, the verification of robustness of watermark in a partial decrypted image is the same as Section 3.3 and Section 3.5. Two examples are shown in Table 12. The partial decrypted image has an advantage. Because the size of the encrypted block is known, the corner points of the encrypted blocks can be used as reference points for perspective correction. As shown in the first example, the image used for detection is perspective-corrected by the four points marked in the captured image. An example of magnitude spectrum of selected detection block is shown following. If we use the encrypted part for watermark extraction, we can crop out the needed part directly. As shown in the second example, we cut out the part marked by the red box in the captured image for detection. Both methods can achieve good performance.

## 4. Discussion

### 4.1. Characteristic of Screen-Cam Robust Watermarking

The screen-cam process causes severe image quality degradation [45]. In other words, we need to improve the robustness of the watermarking algorithm to deal with a screen-cam attack. A robust watermarking algorithm has three mutually restrictive characteristics [70]: robustness, imperceptibility, and watermark capacity. Under these circumstances, commonly, we need to sacrifice some watermark capacity or imperceptibility to meet the screen-cam robust requirements. For example, the length of message sequences in [43,44,65] were only 63, 60, and 94 bits, which are less than normal. Besides, these methods all embedded the message repeatedly to deal with the loss of detailed information during screen-cam process.

In the proposed method, we also employed the above ideas to achieve screen-cam robust of watermark A in a decrypted image. Furthermore, we designed a DFT-based global watermarking algorithm to deal with the loss of detailed information during screen-cam process. As we mentioned in Section 2.2.2, by employing this method, we could select a block larger than one watermark embedded block to contain more detailed information for watermark extraction.

The characteristic of watermark B against screen-cam attack in the encrypted image is special, that is because the encrypted image is a noise-like image. If the encrypted image can be modulated into a noise image similar to the meaningless watermark pattern, this is equivalent to enhancing the perception of the watermark and the robustness is significantly improved. Therefore, it provides the possibility to increase the watermark capacity. Based on this, we can design a QR code-based watermark generation method that contains a message sequence of 620 bits.

### 4.2. Analysis of Joint Encryption and Watermarking Mechanism

How to combine encryption and watermarking technology is a scientific issue. In previous research, the encryption and watermarking worked independently to a certain extent or watermarking was limited by the method itself. The previous joint encryption and watermarking methods were mainly divided into two categories: CEW and RDH-EI.

CEW methods can be further divided into three types [71]. The first one is based on different data fields, which means two independent parts are used for encryption and watermarking respectively [72,73]. Therefore, to some degree, encryption and watermarking work independently. The second type is invariant-based, where the watermark is embedded in a subset that is invariant before and after encryption [11,74]. However, the robustness is also limited by the used invariants. For example, because global histogram statistics are invariable after encryption by scrambling pixel positions, [11] employed a histogram-based watermarking method to achieve CEW. However, the histogram-based method is susceptible to cropping attacks and certainly not applicable for screen-cam attack. The third type of CEW is based on homomorphic encryption, where algebraic operations on the original data can be realized by performing (possibly different) algebraic operations on the encrypted data [75]. Similarly, homomorphic-based CEW is limited by the method itself. Because the algebraic operations that can achieve homomorphism are limited, the corresponding watermarking algorithms that can be designed are also limited.

RDH-EI methods distinguish between content owner and data hider [17], where data hider can only read the reversible watermark but cannot access the encrypted data. Most RDH-EI methods can be divided into two frameworks: vacating room after encryption and reserving room before encryption. Therefore, to some degree, the encryption and watermarking of RDH-EI methods also work independently.

The joint encryption and watermarking mechanism of the proposed scheme is different from CEW or RDH-EI. We embedded the watermark through odd-even quantization and encrypted odd and even to different numerical ranges. In this way, the encryption method could enhance the perceptibility of watermark B in the encrypted image, thereby achieving screen-cam robust. In addition, as we mentioned in Section 2.1.2, the watermarked and encrypted image could effectively avoid the weakness of the proposed encryption algorithm compared to the only-encrypted image. Therefore, this proposed design achieved the mutual cooperation of encryption and watermarking technologies. However, there is no doubt that the design of encryption and watermarking methods are still mutually restricted.

In practical applications, the joint mechanisms of encryption and watermarking are neither superior nor inferior to each other. The joint mechanism needs to be decided according to the requirements of algorithm design. In order to meet more application scenarios and requirements, the joint encryption and watermarking mechanism is worthy of further exploration.

## 5. Conclusions

This paper proposes a joint encryption and screen-cam robust watermarking scheme, which can achieve watermark extraction from both encrypted and decrypted images taken by a smartphone. In watermark embedding and image encryption, first we embed a watermark A with a DFT-based algorithm, then the watermark B was generated based on QR encoding and IDFT to achieve high watermark capacity and error correction ability. After that, watermark B was hidden in the changes caused by embedding watermark A, which can improve imperceptibility and does not affect the effectiveness of watermark A. Finally, a chaotic mapping-based segment encryption algorithm was proposed, which can match with watermark B and enhance its robustness after encryption. With respect to watermark detection from an encrypted image, a frame detection method was utilized to achieve watermark synchronization. With respect to watermark detection from the decrypted image, we used a large size of block and searched the tracking sequence based on NCC coefficients to locate the watermark message. The watermark messages were all extracted from the noise component with a local statistic feature. The proposed scheme is proved to have a high robustness to the screen-cam process before and after decryption, and also has a remarkable performance against common image processing attacks after decryption.

## Figures and Tables

**Table 1:** Resynchronizing Result of se with 5 × 5 Size.

$T_{1}$	Shooting Distance (cm)			
	10	20	30	40
0.55	×	√	√	√
0.6	×	√	√	√
0.65	√	√	√	√
0.7	√	√	√	√
0.75	√	√	√	×
0.8	√	×	×	×

**Table 2:** Resynchronizing result of se with 7 × 7 Size.

$T_{1}$	Shooting Distance (cm)			
	10	20	30	40
0.55	×	√	√	√
0.6	√	√	√	√
0.65	√	√	√	×
0.7	√	√	×	×
0.75	√	×	×	×
0.8	√	×	×	×

**Table 3:** Resynchronizing result of se with 9 × 9 Size.

$T_{1}$	Shooting Distance (cm)			
	10	20	30	40
0.55	√	√	√	×
0.6	√	√	√	×
0.65	√	√	×	×
0.7	√	×	×	×
0.75	√	×	×	×
0.8	×	×	×	×

**Table 4:** Average BER under different shooting conditions in watermark B extraction.

Shooting Horizontal Angle (Left)	Shooting Distance			
	10 cm	20 cm	30 cm	40 cm
0°	1.8/597	2.3/597	3/597	6.4/597
15°	2.8/597	3.2/597	4.3/597	6.7/597
30°	3.3/597	3.4/597	5/597	8.1/597
45°	3.6/597	6.8/597	8.8/597	11.5/597

**Table 5:** Examples of automatically extraction result of watermark B.

Shooting Condition	Captured Photo	Extracted Block	Magnitude Spectrum	BER
Shooting distance: 10 cm. Shooting angle: 45°	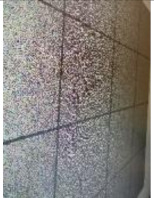	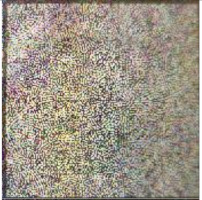	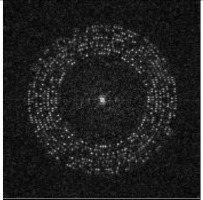	2/597
Shooting distance: 20 cm. Shooting angle: 45°	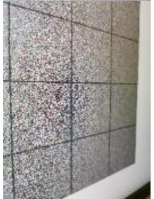	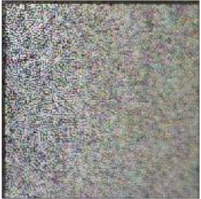	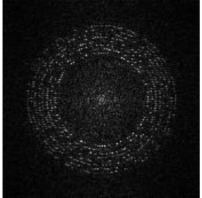	5/597
Shooting distance: 30 cm. Shooting angle: 45°	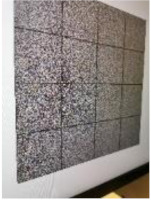	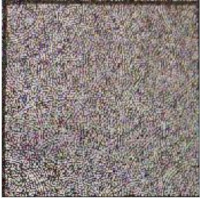	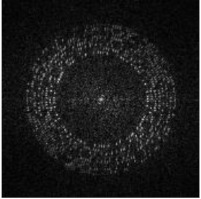	8/597
Shooting distance: 40 cm. Shooting angle: 45°	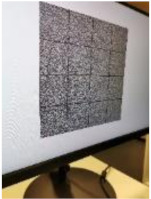	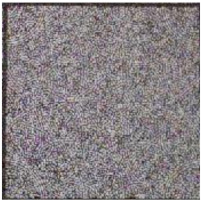	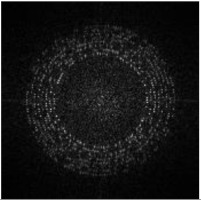	10/597

**Table 6:** Examples of extraction result of watermark B with manual operation.

Captured Image	Manual Cropping and Scaling	Extracted Block	Magnitude Spectrum	BER
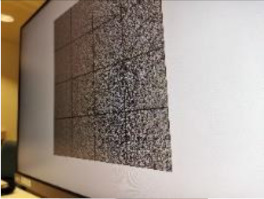	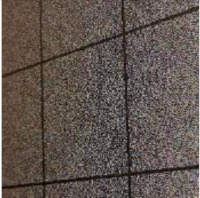	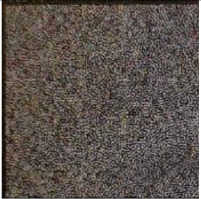	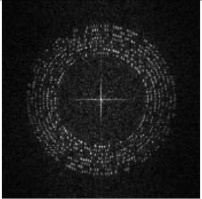	6/597
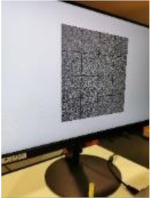	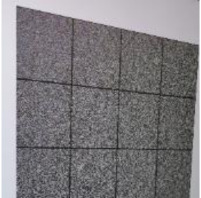	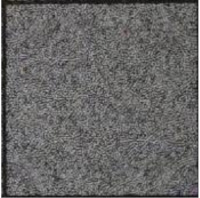	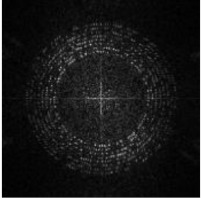	7/597
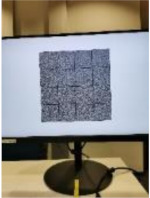	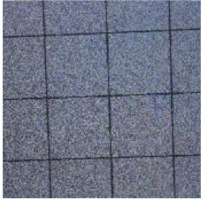	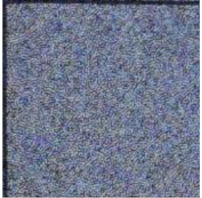	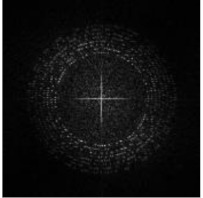	15/597

**Table 7:** Watermarked images generated by different methods.

Methods	Pramila et al. [32]	Fang et al. [43]	Chen et al. [44]	Proposed
Image	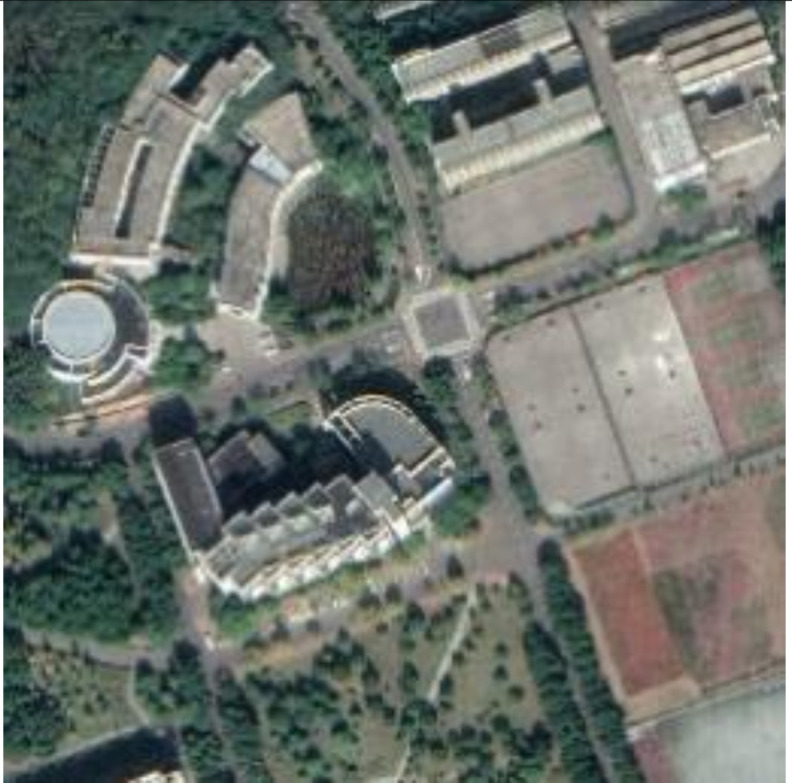	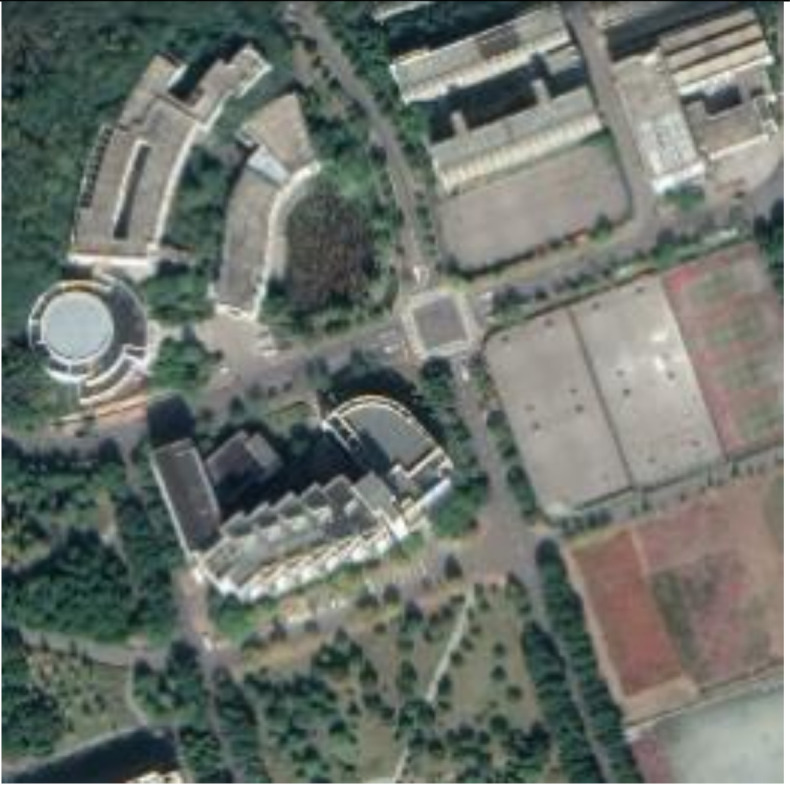	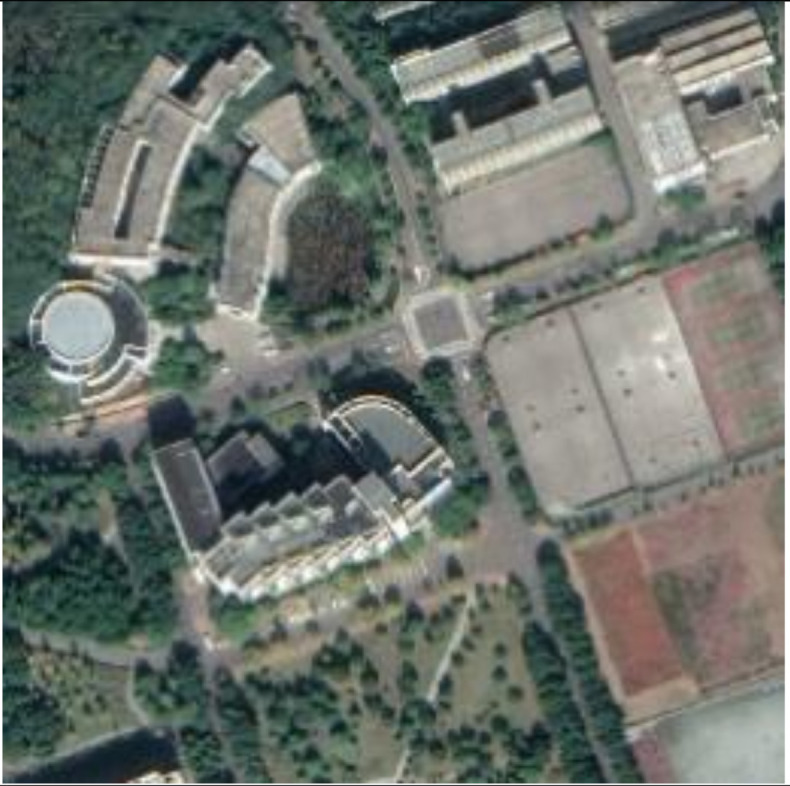	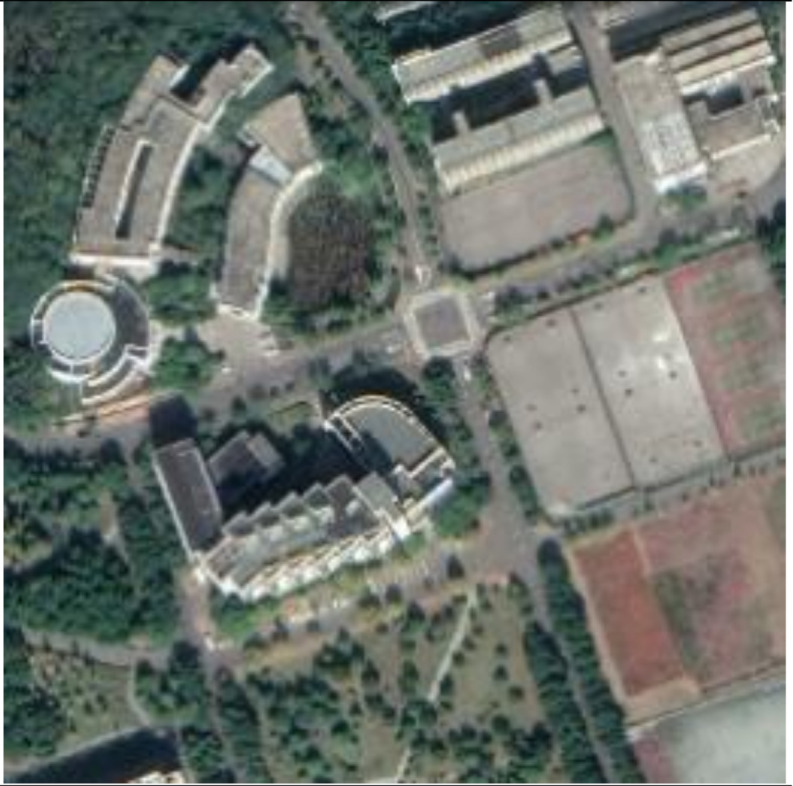
PSNR (dB)	40.3206	40.2210	40.7022	40.8513
SSIM	0.9589	0.9539	0.9661	0.9672

**Table 8:** Average bit error rate (BER) under different common attacks.

Attacks	BER			
	Primila et al. [32]	Fang et al. [43]	Chen et al. [44]	Proposed
JPEG 40	43.91%	0.16%	0.11%	0.16%
JPEG 30	49.69%	4.76%	0.86%	1.11%
JPEG 20	50.31%	23.02%	5.59%	6.98%
Scaling 200%	49.84%	49.84%	0%	0%
Scaling 50%	47.66%	50.16%	−	0%
Scaling 40%	50.31%	51.43%	−	−
Rotation 10° + cropping	52.81%	49.68%	0.00%	0.00%
Rotation 15°+ cropping	48.75%	48.25%	0.00%	0.00%
Rotation 30°+ cropping	49.53%	52.06%	−	0.00%
Median filter 3 × 3	4.69%	0.00%	1.18%	0.00%
Median filter 4 × 4	9.69%	0.79%	10.32%	6.51%
Gaussian Noise (0.005)	19.06%	5.08%	7.10%	4.92%
Gaussian Noise (0.01)	28.59%	11.27%	20.54%	6.83%
Salt & Pepper (0.05)	35.00%	14.76%	2.58%	0.16%
Poisson	31.72%	3.33%	1.94%	0.63%
Speckle	39.22%	15.08%	1.94%	0.79%
Sharpening	0.63%	0.48%	1.08%	0.95%
Linear adjustment	0.63%	0.00%	0.00%	0.00%
Histogram equalization	0.47%	0.00%	0.00%	0.00%

**Table 9:** Average EBR with different shooting conditions.

Horizontal Angle (Left)	Shooting Distance							
	30 cm	40 cm	50 cm	60 cm	70 cm	80 cm	90 cm	100 cm
0°	1.3/63	0.8/63	1.2/63	0.7/63	1.3/63	1.2/63	2.6/63	3.7/63
15°		1.3/63	1.4/63	0.9/63	1.5/63	1.7/63	2.3/63	8.8/63
30°		3.3/63	2.3/63	1.6/63	2.2/63	2.6/63	5.4/63	11.5/63
45°		2.4/63	3.3/63	3.6/63	3.9/63	5.8/63	/	/
60°		3.0/63	3.6/63	4.9/63	11.7/63	/	/	/

**Table 10:** Examples of NNU recovered from different captured images.

Horizontal Angle (Left)	Shooting Distance							
	30 cm	40 cm	50 cm	60 cm	70 cm	80 cm	90 cm	100 cm
0°	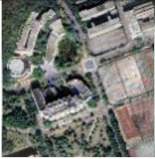	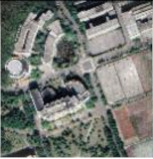	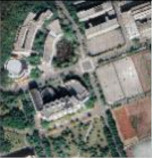	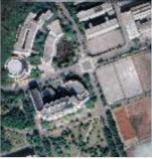	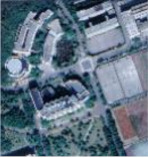	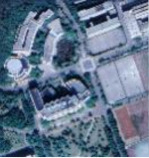	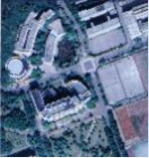	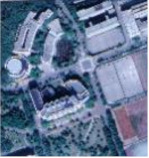
BER	0/63	1/63	2/63	0/63	0/63	0/63	0/63	3/63
15°		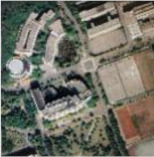	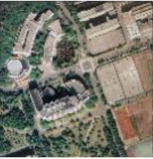	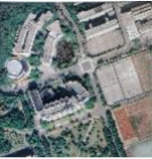	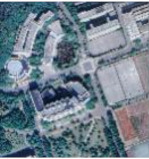	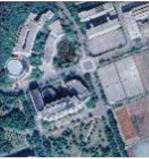	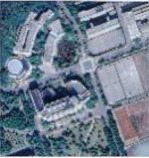	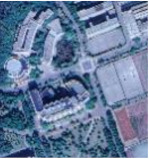
BER		1/63	0/63	0/63	1/63	1/63	5/63	11/63
30°		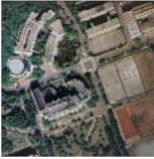	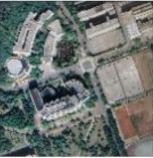	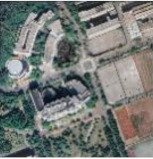	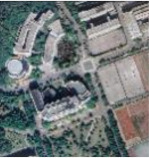	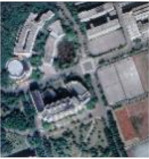	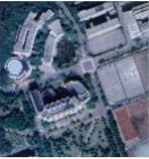	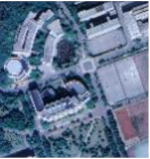
BER		2/63	1/63	2/63	1/63	1/63	4/63	8/63
45°		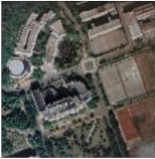	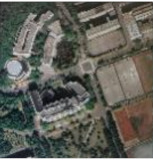	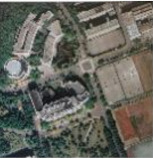	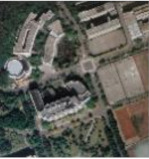	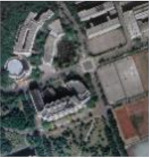	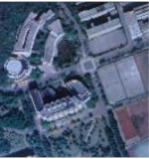	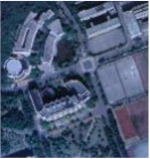
BER		1/63	1/63	3/63	1/63	5/63	/	/
60°		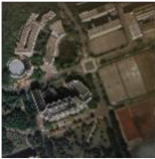	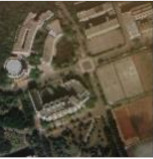	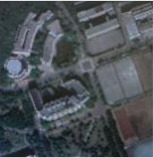	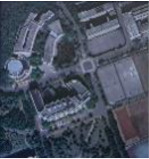	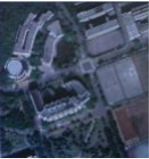	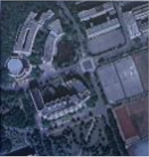	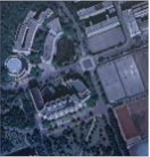
BER		3/63	5/63	6/63	9/63	/	/	/

**Table 11:** Examples of handhold shooting.

Handhold Scenarios	Example 1	Example 2	Example 3	Example 4
Captured image	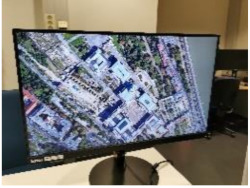	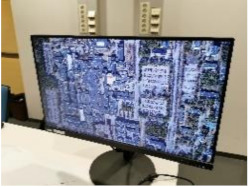	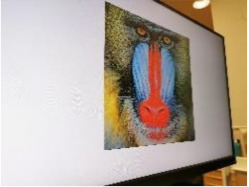	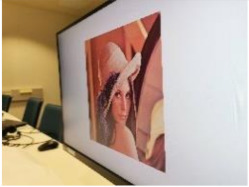
Recovered image	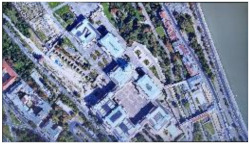	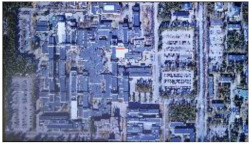	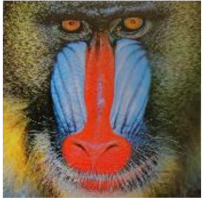	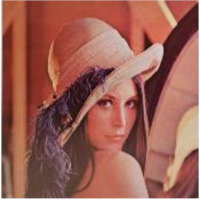
BER	3/63	5/63	1/63	2/63

**Table 12:** Watermark extractions from partial decrypted image.

Captured Image	Used for Detection	Magnitude Spectrum	BER
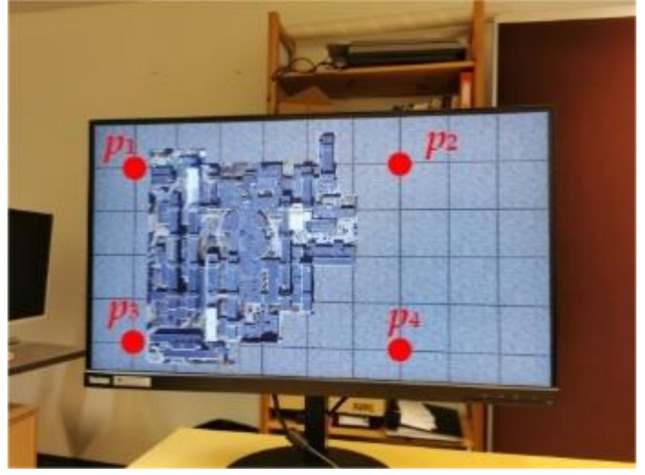	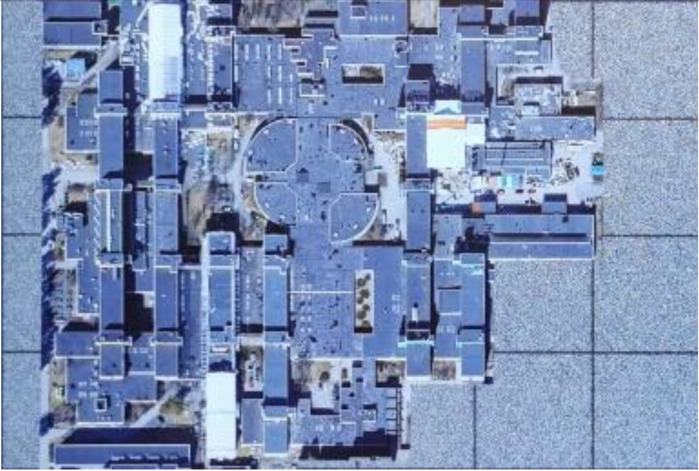	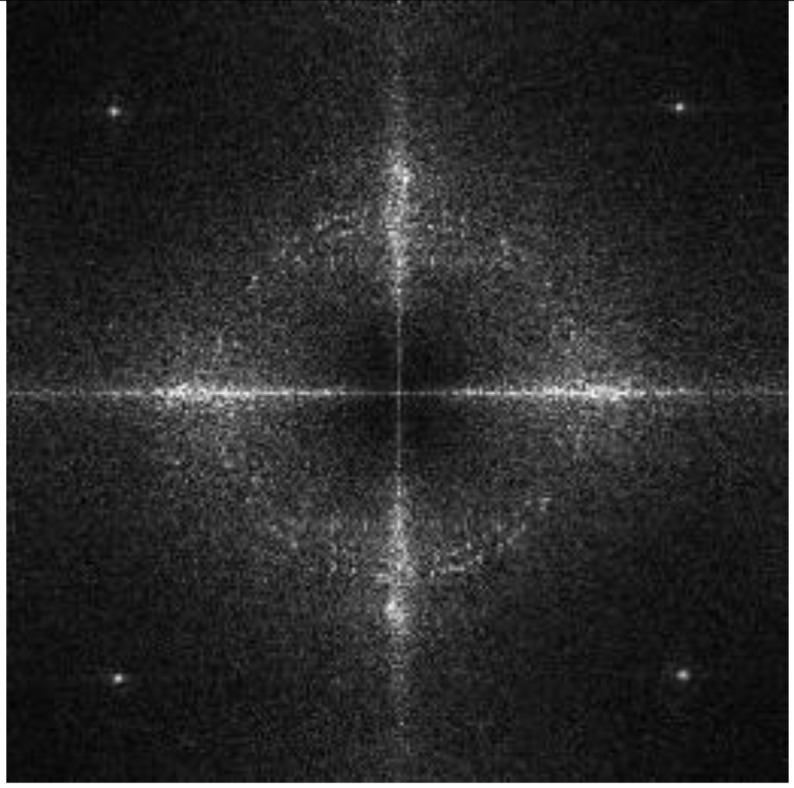	4/63
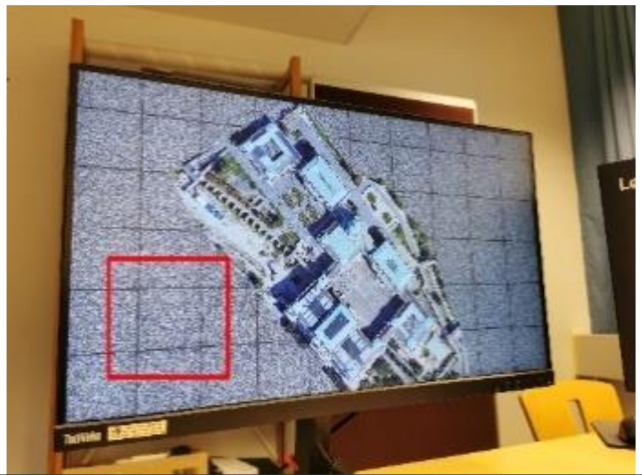	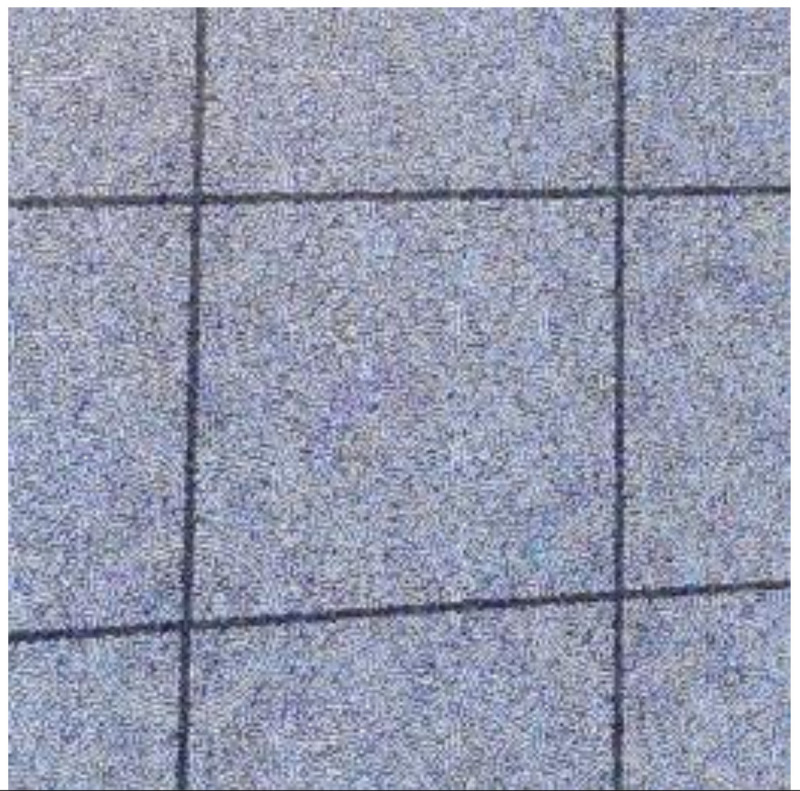	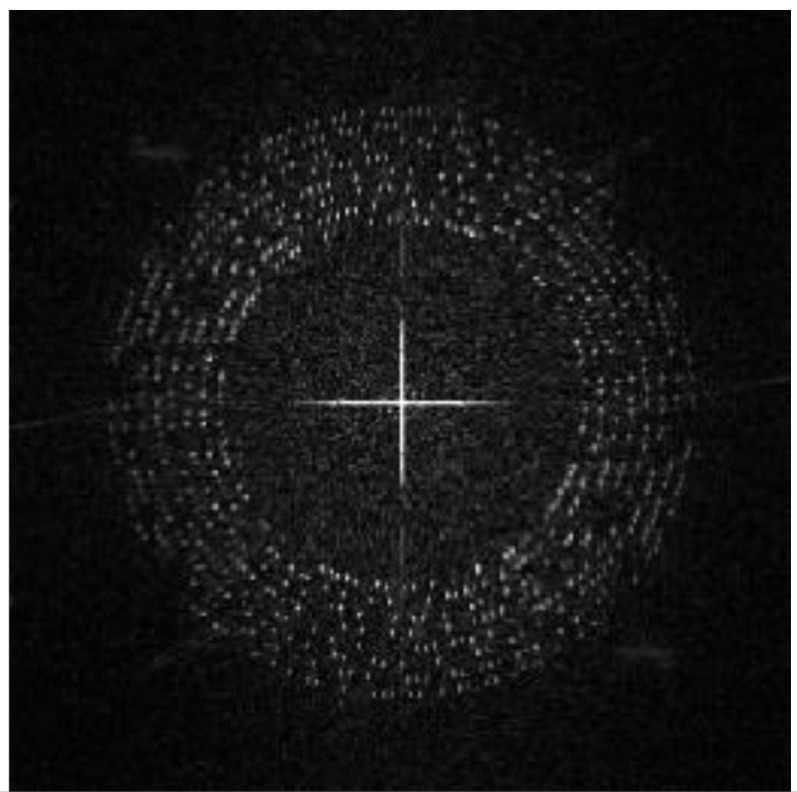	3/597

## Data Availability

Data is contained within the article.
